# Probiotic as a Novel Treatment Strategy Against Liver Disease

**DOI:** 10.5812/hepatmon.7521

**Published:** 2013-02-25

**Authors:** Abbas Ali Imani Fooladi, Hamideh Mahmoodzadeh Hosseini, Mohammad Reza Nourani, Soghra Khani, Seyed Moayed Alavian

**Affiliations:** 1 Applied Microbiology Research Center, Baqiyatallah University of Medical Sciences, Tehran, IR Iran; 2 School of Pharmacy, Student's Research Committee, Tabriz University of Medical Sciences, Tabriz, IR Iran; 3 Tissue Engineering Division, Chemical Injury Research Center, Baqiyatallah University of Medical Sciences, Tehran, IR Iran; 4 Department of Biochemistry, Pasteur Institute of Iran, Tehran, IR Iran; 5 Baqiyatallah Research Center for Gastroenterology and Liver Diseases, Baqiyatallah University of Medical Sciences, Tehran, IR Iran

**Keywords:** Probiotics, Liver Cirrhosis, Nonalcoholic Fatty Liver Disease, Hepatic Encephalopathy, Liver Diseases, Alcoholic, Hepatitis, Carcinoma, Hepatocellular

## Abstract

**Context:**

A symbiotic relationship between the liver and intestinal tract enables the healthy status of both organs. Microflora resident in intestinal lumen plays a significant role in hepatocytes function. Alterations to the type and amount of microorganisms that live in the intestinal tract can result in serious and harmful liver dysfunctions such as cirrhosis, nonalcoholic fatty liver disease, alcoholic liver disease, and hepatic encephalopathy. An increased number of pathogens, especially *enterobacteriaceae, enterococci*, and *streptococci* species causes the elevation of intestinal permeability and bacterial translocation. The presence of high levels of lipopolysaccharide (LPS) and bacterial substances in the blood result in a portal hypertension and ensuing hepatocytes damage. Several methods including the usage of antibiotics, prebiotics, and probiotics can be used to prevent the overgrowth of pathogens. Compared to prebiotic and antibiotic therapy, probiotics strains are a safer and less expensive therapy. Probiotics are "live microorganisms (according to the FAO/WHO) which when administered in adequate amounts confer a health benefit on the host”.

**Evidence Acquisitions:**

Data from numerous preclinical and clinical trials allows for control of the flora bacteria quantity, decreases in compounds derived from bacteria, and lowers proinflammatory production such as TNF-α, IL-6 and IFN-γ via down-regulation of the nuclear factor kappa B (NF-κ B).

**Results:**

On the other hand, probiotic can reduce the urease activity of bacterial microflora. Furthermore, probiotic decreases fecal pH value and reduces ammonia adsorption. In addition, the serum level of liver enzymes and other substances synthesized by the liver are modulated subsequent to probiotic consumption.

**Conclusions:**

According to our knowledge, Probiotic therapy as a safe, inexpensive and a noninvasive strategy can reduce pathophysiological symptoms and improve different types of liver diseases without side effects.

## 1. Context

Several endogenous and exogenous factors have negative consequences on the liver and cause destruction of the hepatic cells, thus leading to different kinds of liver diseases. Primary abnormal conditions such as alteration of microbial populations in intestinal tracts, have been known as common risk factors for obesity and diabetes type I (1). In addition, the gastrointestinal system has a close association with the liver. The gut, especially in the large intestine, contains large numbers of microorganisms. Almost 300 to 500 different kinds of species reside in it (2). The number of bacteria and their genes are more than the cells and genes belonging to each person. According to Neish study, 10^9^ colony forming units (CFU)/ml and 10^12^ CFU/ml of bacteria may found in the terminal ileum and colon. Moreover, gram negative bacteria, and anaerobes are dominant species in the intestinal lumen which are estimated to be 100 to 1000 times more than aerobic ones. *Bacteroides, Porphyromonas, Bifidobacterium, Lactobacillus, Clostridium* and *Escherichia coli (E. coli)* are the most frequent ones (3). However, in each person, the pattern of microorganism population is unique and different (4). Indeed, microflora in the human gut lumen is a dynamic and complicated ecosystem which is capable of restoring itself and remains constant in most physiological conditions. In healthy individuals, the intestinal microflora contributes to various processes which affect the intestinal functions. Preparation of some substances such as nutrients, vitamin K, folate, short chain fatty acids, and peroxides are some examples. In addition, bacteria can digest unabsorbed sugars including lactose together with alcohol, and produce short chain fatty acids which mucosa and enterocytes use as energy source. Modulation of the growth, proliferation, and differentiation of epithelial cells located in the intestine are other roles of these fatty acids (5). Furthermore, this complex ecosystem plays a key role to enhance immunity against pathogens entry to the body from external environment. Improvement of the host`s defense system depends on the presence and activation of receptors such as TLRs (toll-like receptor), which can recognize the highly conserved pathogen–associated molecular patterns (PAMPs) (3, 6). Activation of the TLR promotes the production of cytokines, chemokines, and antimicrobial agents through the induction of NF-κB signaling (7, 8). Since the gut mucosa has a unique lymphoid tissue named as GALT (gut associated lymphoid tissue), interaction between the intestinal bacteria and this immune system leads to stimulation of adaptive type of immunity against PAMPs. Microorganisms existing in the microflora population were colonized in, and adhere to the intestinal mucosa, and prevent the growth and colonization of pathogen bacteria. Furthermore, secretion of specific bacteriocins by microflora inhibits the overgrowth of pathogens (5). Any disruption in the amount and composition of gut microflora results in a disturbance to the intestinal homeostasis. Increase in the pathogen population due to malfunction of microflora may lead to severe systematic infections (9). A close anatomical and functional relationship between two organs, the gut and the liver, is known as the gut-liver axis. Most blood supplied to the liver is supplied by the intestine via the portal vein. Blood circulated in the portal vein transfers various toxic compounds such as bacteria and their derivatives, substances produced by microflora including ethanol, ammonia, and acetaldehyde for filtration by liver and modulates kupffer cells activity and cytokine production. The increase of PAMPs and accumulation of metabolites in the liver can cause the liver harm. In return, the liver secretes bile acids to the intestine and modulates its activities (10). Alterations in the type and amount of microorganisms are important elements in the dysfunctions of the liver. Bacterial translocation (BT) occurs and there is an increased amount of microorganisms in the intestinal tract. The BT phenomenon is the migration of bacteria across the intestinal wall to extraintestinal site such as the mesenteric lymph nodes (11). Three factors including characteristics and nature of bacteria, functional properties of the intestinal wall, and local immunity affect the level of BT (12, 13). Some gram-negative species belonged to *enterobacteriaceae, enterococci*, and *streptococci* families are the most frequent bacteria which contribute to BT in patients with cirrhosis. These members, especially some strains of *E. coli*, could adhere to the mucosal surface on intestinal walls and pass across them efficiently. In contrast to higher levels of anaerobic species in microflora, they rarely participate in BT. High amounts of these anaerobic bacteria play a significant role in limiting the growth of species population which can translocate from the intestinal barrier (11). In addition, a multifactorial hypomotility consequent to adrenergic activity is one of the causes of overgrowth. It increases nitric oxide (NO) synthesis which leads to an impaired intestinal structure due to oxidative stress and portal hypertension (14-16). Furthermore, several previous studies reported that permeability changes in the intestine are able to enhance BT. The intestinal wall comprises mucosa, microvilli, enterocytes, and tight junctions attached to the apical surface of enterocytes (17-19). Abnormal changes in any part influence its permeability. Portal hypertension results in mucosa thickness via dilatation mucosa, the lamina propria edema, and proliferation of fibromuscular (20). In addition, increased lipid peroxidation, and oxidative stress in the brush cell membrane are effective in BT promotion (21). Tight junctions are second barriers to inhibit paracellular translocating of bacteria. Integrity impairment of tight junction structure leads to increased intestinal permeability (17). Another important factor interfering with intestinal permeability is a high concentration of toxic acetaldehyde produced via metabolism of ethanol due to large bowel bacteria overpopulation (22, 23). Increasing BT leads to the presence of a high quantity of LPS (24), and bacterial DNA (25) in circulation. Lack of hepatic clearance of these components from circulation is enhanced due to portal hypertension in liver disease. LPS as an endotoxin located in gram negative bacteria cell wall was recognized by TLR expressed on macrophages and stimulate proinflammatory cytokine secretions. Additionally, high amounts of bacterial DNA, and their derivatives induce the production of TNF-α, IL-2, IL-6, IL-12, inducible nitric oxide synthesis, and nitric oxide (25, 26). In the liver, the extensive attachment of LPS to CD4/TLR4 induces high amounts of LPS-binding protein (24). Inefficient local immunity was demonstrated in liver disease; patients with cirrhosis in particular. The potent mechanism is depression in the activity of kupffer cells, and the reticular endothelium system which plays a significant role in defense against infected bacteria (27). Bile acids, secretary IgA, mucine, defensin, lysozyme and phospholipase A2 are agents which modulate bacterial growth in the intestine tract (28).

Our objective was to explain the role of probiotics as a bacteriotherapy strategy for treatment of some liver diseases.

## 2. Evidence Acquisition

### 2.1. Probiotic

According to the FAO/WHO definition, probiotics are as “Live microorganisms which when administered in adequate amounts confer a health benefit on the host” (29). At the beginning of 20^th^ century, Elie Metchnikoff introduced a novel hypothesis about the health effects of probiotics. He claimed that the consumption of fermented milk products led to the health and longevity of Bulgarian peasants. Moreover, he stated that the organisms that lived in local yogurt were able to protect the intestine from the destructive effects of other pathogenic bacteria (30). Ideal probiotic strains have special properties such as resistance to bile, hydrochloric acid, and pancreatic juice; the ability to tolerate stomach and duodenum conditions and gastric transport; stimulation of the immune system, thereby improving intestinal function via adhering and colonizing the intestinal epithelium. In addition, probiotic strains competed with pathogens and modulated permeability, produced lactic acid, and exhibited anticarcinogenic and antipathogenic activity. Furthermore, these strains must be able to survive during the production processes and storage and still exert considerable healthful outcomes (31). *Lactobacillus, Bifidobacterium, Escherichia, Enterococcus, Bacillus, Streptococcus*, and some fungal Saccharomyces strains have been known as probiotics (32, 33). A powder, liquid, gel, paste, granule, capsule, sachet, and several kinds of food are available commercial products containing probiotics (34). It has been confirmed that 10^8^ to 10^11^ CFU per day can demonstrably show the healthy effects of the probiotics (34, 35). Properties and effective actions of each probiotic are unique; therefore it is necessary to select a desired strain for treatment of each disorder. Several studies and clinical trials have been performed to assess the effects of various strains of probiotics for treating or preventing some diseases including special kinds of diarrhea, inflammatory bowel disease, cancer, Helicobacter pylori infection, vaginosis, hepatic disease allergy, lactose intolerance, high cholesterol levels, colitis, modulation of immune system, and several other abnormalities. Khani *et al.* published a useful review to shed light on these subjects (34).

### 2.2. Gut Microflora in Pathogenesis of Liver Disease

#### 2.2.1. Nonalcoholic Fatty Liver Disease (NAFLD)

NAFLD is the most prevalent liver disease globally. It occurs in all age groups from children to adults (36). NAFLD includes an extensive range of disorders from steatosis to nonalcoholic steatohepatitis (NASH). A broad spectrum of histological manifestations including macrovesicular steatosis, liver cirrhosis, portal hypertension, and hepatocellular carcinoma are observed in NAFLD. A high amount of lipid storage in hepatocytes increases liver transaminase, and accumulation of necroinflammation components are indicators of a diseased condition (37). NAFLD is related to diabetes, insulin resistance, and obesity. Overall, complications of the metabolic syndrome are observed in these patients (36). Recently, two theories have been introduced regarding the manifestations of NASH. The first theory states that insulin resistance enables the transportation of fatty acids from adipose tissues to the liver. In the second one, excessive harmful compounds such as bacterial LPS, inflammatory inducer substances, and different substrates as energy sources (ethanol, short fatty acids) due to the overgrowth of gut microflora are known as causal factors (38). Overproduction of ethanol, endotoxin, and BT phenomena followed by abnormal growth of gram negative bacteria, stimulation of innate immunity in the liver and induction of hepatic oxidative damages result in liver injuries and cirrhosis (39).

#### 2.2.2. Alcoholic Liver Disease (ALD)

ALD is a cause of a high rate of morbidity and mortality worldwide. Alcoholic steatohepatitis (ASH), and severe ALD manifest in approximately 30% of heavy drinkers (40). Therefore, other factors contribute to the emergence of ASH. Results from various in vivo studies performed on animals and humans reported that endotoxin produced by bacteria living in the bowel tracts functions as a cofactor. Furthermore, a high level of endotoxin has been seen in the plasma of these patients (41-45). Hyperpermeability of the intestine following by alcohol consumption leads to endotoxemia, which is filtrated by the liver and triggers the proinflammatory pathways for causing ASH. In addition, the leakiness of gut activates NF-kB transcription and overexpression of nitric oxide synthesis. Increased synthesis of NO results in oxidative stress in hepatocytes (46, 47). Alcohol consumption over a long period elevates the growth of gram negative bacteria and increases the amount of *bifidobacteria* and *lactobacilli*.

#### 2.2.3. Cirrhosis

Cirrhosis, a vascular disease, is recognized by attributes such as portal hypertension, and hyperdynamic syndrome (10, 48). Similar to most liver diseases, the lack of equilibrium in gut normal-flora, and impairment of the intestinal barrier cause endotoxemia, a high level of proinflammatory cytokines, and NO synthesis induction (49, 50). Overgrowth of gut microflora, BT, and endotoxemia found in patients with cirrhosis supported the use of vascular shunts. As described before, these complications are accompanied with stimulation of inflammation and oxidative damage in liver which cause hepatocytes injuries (51).

#### 2.2.4. Primary sclerosing cholangitis (PSC)

PSC is an autoimmune liver disease which involves bile ducts in and out of the liver. Cholestatic features of bile ducts are a result from progressive obliterative fibrosis. Although a close association between PSC and inflammatory bowel disease has been reported (52), however, its pathogenesis remains unknown. Immune and nonimmune mechanisms are suggested for the pathogenesis of PSC. There is a substantial amount of evidence that the lymphocytes located in the gut play a critical role for emerging PSC. On the other hand, bacteria residing in the gut may be a part of the cause of PSC through nonimmune routs (53). These microorganisms are able to release toxic compounds (54). Since the administration of antibiotics is an appropriate treatment for some patients with PSC (54, 55), there is a possibility indicating the role of bacterial flora together with intestinal inflammation in the pathogenesis of PSC.

#### 2.2.5. Hepatic Encephalopathy (HE)

HE is a harmful complication following acute and chronic liver disease which occurs in at least 50% to 70 % of patients with cirrhosis (56, 57). This is a serious secondary neuropsychiatric syndrome which appears as a result of multi factors. Production of ammonia by the gut flora, and its release to the portal system are known as key factors to disrupt the central nerve system, and lead to hepatic encephalopathy (58).

#### 2.2.6. Viral Hepatitis

Some viruses like hepatitis B and C virus (HBV and HCV) are known as causative agents leading to long term hepatocellular injury. High frequency of these viruses was reported in Iranian population (59). The plasma level of endotoxin increases in patients with HBV and HCV. Furthermore, high amounts of the proinflammatory cytokines described above and the necrosis reported in these patients cause liver damage in the longer term (60, 61). Results from a study of lactitol effect on amount of endotoxin in patients with HBV and HCV blood, showed that the alleviation of endotoxemia could be achieved by increasing bifidobacteria and lactobacillus numbers and avoiding the growth of pathogens (62).

#### 2.2.7. Hepatocellular Carcinoma (HCC)

HCC is the most prevalent primary liver tumor which occurs after cirrhosis with a high frequency. Many risk factors such as a presence of viral antigens in chronic hepatitis B virus infections, carcinogenic mycotoxins, and compounds which produce reactive oxygen species alter molecular pathways in hepatocytes, in particular gene expression. For instance, aflatoxin, a strong mycotoxin, can attach to G nucleotides in the P53 gene and convert it to T and causes reduction of P53 transcription. In addition, inhibitory effects of aflatoxin on c-myc and bcl2 result in cell proliferation, and cancer progression (63-66).

### 2.3. Probiotic Mechanisms in Liver Disease

**Figure 1:**
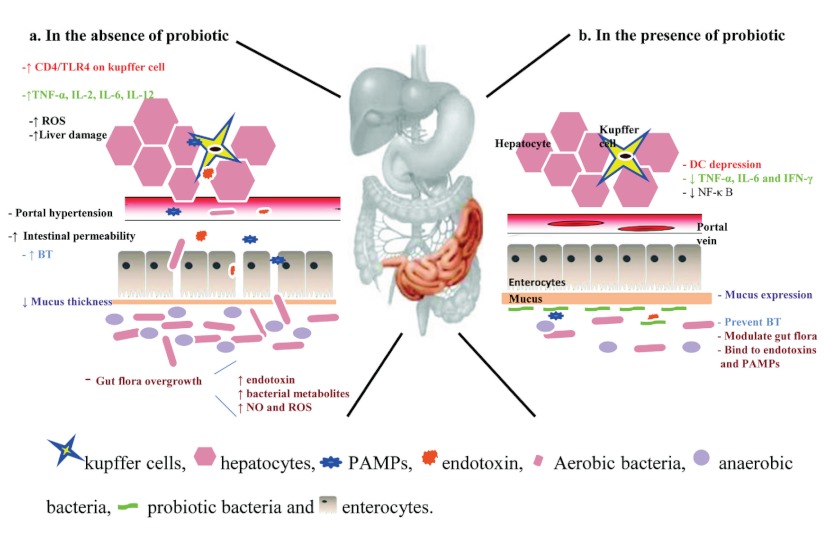
a) Schematic Illustration of Multifarious Mechanisms Involved in Liver Diseases and b) Probiotic Influences on Them

The beneficial effects of probiotics on liver health were observed in several studies. The primary mechanisms reported in these studies are the changes in gut functions (Figure 1). Consumption of probiotics could allow *enterobacteriaceae* through the competitive inhibition. Developing nutritional practices, mucosal barrier repairing, apoptosis prevention due to providing of short chain acids, and improving intestinal epithelial viability are other probiotic effects which stabilize physiological luminal permeability together with lowering ammonia adsorption (67). These functions alleviate tight junction disturbance by pathogens (68), and are essential agents for lowering BT. Induction of anaerobes and gram positive bacteria growth, limiting gram negative bacteria, and preventing pathogens adherents are other antitranslocation effects of the probiotics (69). Controlling flora bacteria quantity can lead to decreased endotoxins and other toxic compounds derived from bacteria such as ethanol, phenol, indoles which cause injury to the liver. Decreased levels of these substances in the liver result in lowering of proinflammatory production such as TNF-α, IL-6, and IFN-γ via down-regulation of the NF-κ B (70).On the other hand, they can depress urease activity of microflora bacteria followed by ammonia production and release in to the portal system. Furthermore, probiotic decreases fecal pH value and reduces ammonia adsorption (71).

### 2.4. Probiotic as a Treatment Strategy of the Liver Disease

#### 2.4.1. NAFLD

Although NAFLD is known as a prevalent type of liver disease globally, the preferred therapy method has not been established. To conquer the overpopulation of pathogens, antibiotic therapy is the current strategy. Polymixin B and metronidazole are antibiotics which are currently used in these patients for ameliorating liver functions. However, it is not an appropriate therapy because of the unspecific ramifications to any bacteria (72). The usage of probiotic, prebiotic and symbiotic bacteria is recommended since these bacteria have the capacity to modulate microflora overpopulations and their consequent effect on the liver. In spite of the presence of various experimental studies in this field and positive effects of probiotic on induced liver steatosis (Table 1), the recognition of this strategy for treating or even preventing NAFLD is still complicated. Two subjects are relevant in this context. Firstly, the species of probiotics used in these studies varied. Most of these experiments were performed on the effects of VSL#3 administration which itself contains a various species such as *Streptococcus thermophilus, Bifidobacterium berve, Bifidobacterium longum, Bifidobacterium infanti, Lactobacillus acidophilus, Lactobacillus plantarum. Lactobacillus casei*, and *Lactobacillus bulgaricus* (73-78) and determination of the effect of each species is unclear. Furthermore, most of them were tested on animal models and insufficient human clinical trials have been performed (Table 1). Therefore approving these bacteria as a treatment protocol requires several large scale clinical trials.

**Table 1:** A list of Multifarious Studies on NAFLD

Studies	Participants/Duration	Treatment	Outcome	Reference
**Animal models**	Ob/ob mice fed HFD / 4 w	VSL # 3	hepatic FA content, ALT level, activity of Jun N-terminal kinase, NF-κB and fatty acid β-oxidation, improved hepatic IR, and NAFLD histology	(74)
**Animal models**	mice fed HFD / 4 w	VSL # 3	Ameliorate hepatic NK cell depletion, steatosis, IR and inflammation, cholesterol and TG in the liver and plasma	(76)
**Animal models**	mice fed HFD / 8 w	*Lactobacillus rhamnosus PL60*	liver steatosis, improved histological steatosis manifestation	(83)
**Animal models**	Rats fed HFD & HCD / 6 w	*Bacillus polyfermenticus SCD*	LDL, cholesterol and triglycerides	(84)
**Animal models**	Rats fed high-fructose diet / 8 w	*Lactobacillus acidophilus and Lactobacillus casei*	oxidative stress and ameliorate IR in liver	(85)
**Animal models**	Rats fed HCD / 5w	*Lactobacillus plantarum MA2*	cholesterol and triglycerides	(86)
**Animal models**	Rats fed HFD / 4w	VSL # 3	Improved the hepatic inflammatory, steatotic, peroxidative factors, serum aminotransferase levels	(73)
**Animal models**	mice fed MCD / 9 w	VSL # 3	only improved liver fibrosis without effect on statosis and inflammation	(78)
**Animal models**	Rats/8 w	*Lactobacillus paracasei F19*	hepatic inflammation, steatosis and fibrosis, innate inflammatory cytokines	(87)
**Pilot study**	10 NASH patients / 2 m	*L. acidophilus, L. bulgaricus, B. lactis, B. bifidus, L. plantarum, l. breve, L. casei, L. salivarus, L. rhamnosus vs* FOS and vitamin	improved liver damage and liver function test ALT, AST, and GGT activity Ameliorate MDA and 4-HN plasma level	(88)
**Open pilot**	22 patients / 3 m	VSL # 3	improved liver damage and liver function test ALT, AST, and GGT activity Ameliorate MDA, and 4-HN plasma level	(75)
**OL, pilot trial**	4 patients / 4 m	VSL # 3	liver fat After washout time, no effects in blood or clinical parameters	(77)
**R, DB, PC**	28 patients / 3 m	*L. bulgaricus, S. thermophilus*	ALT, AST, and GGT activity	(89)

Abbreviation: ALT, alanin aminotransferase; AST, aspartate aminotransferase; DB, double-blind; d, day; FOS, fructo-oligosaccharide.synbiotic; GGT, gamma glutamyl transferase, m, month; HCD, high-cholesterol diet; HFD, high fat diet; MDA, malondialdehyde; 4-HN, 4-hydroxynonenal; OL, open labeled; PC, placebo-controlled; R, randomized; w, week.

#### 2.4.2. ALD

As already described, quantitative and qualitative alterations in the intestinal microflora are related to alcohol intake. In contrast to knowledge about most causative factors involved in the ALD pathogenesis, unfortunately, appropriate treatment of these patients is inconclusive as of yet. Since probiotic strains can modulate the gut and immune systems, it is suggested that probiotics can be administered to relieve ALD symptoms. Data from the studies designed to evaluate the role of probiotic supplementation in patients and animal models of ALD is summarized in Table 2. Improving the liver variables such as serum liver enzyme (alanin aminotransferase, aspartate aminotransferase and gamma glutamyl transferase), and total bilirubin, ameliorating hepatic inflammation and histological grade are results from probiotic therapy in these studies.

**Table 2:** Studies on Animal and Human Subjects with ALD

Study models	Participants/Duration	Treatment	Outcome	Reference
**Animal models**	Rat fed ethanol / 1 m	*Lactobacillus GG*	Any hepatic pathologic alterations in induced by alcohol, endotoxin level	(90)
**Animal models**	Rat fed ethanol / 10 w	*Lactobacillus GG*	Gut permeability, decreased hepatic and intestinal oxidative stress and inflammation	(91)
**Animal models**	Rat fed ethanol / 10 w	*Lactobacillus GG *and oats	Preventing alcohol-induced dysbiosis	(92)
**Animal models**	Mice fed ethanol / 35 d	heat-killed* Lactobacillus brevis SBC8803*	ALT, AST, TG, and cholesterol level Inhibit overexpression of TNF-a, SREBP-1, SREBP-2	(93)
** DB, PC**	39 patients / 42 d	*E. coli Nissle*	Improving intestinal colonization, and restore physiological Microflora in faces, blood endotoxin level, Child-Pugh score.	(94)
**Open label study**	12 patients / 4 w	*Lactobacillus casei Shirota*	Restore neutrophil function, Ex vivo endotoxin-stimulated levels of sTNFR1, sTNFR2 and IL10 normalized TLR4 expression	(95)
**Open label study, RCT**	66 patients / 5 d	*B. bifidumand Lactobacillus plantarum 8PA3* vsabstinence plus vitamins	Bifidobacteriaand Lactobacilli, ALT, AST , LDH and total bilirubin	(96)
**Open pilot study**	20 patients / 3 m	VSL#3	Improving MDA, 4-HN, ALT, AST, GGT, TNF-a, IL-6, and IL-10 levels	(75)

Abbreviation: ALT, alanin aminotransferase; AST, aspartate aminotransferase; DB, double-blind; d, day; GGT, gamma glutamyl transferase; MDA, malondialdehyde; 4-HN, 4-hydroxynonenal; m, month; OL, open labeled; PC, placebo-controlled; RCT, randomized clinical trial; SREBP-1, Sterol regulatory element-binding protein-1; w, week.

#### 2.4.3. Cirrhosis

Bacteriotherapy with probiotic strains in patients with cirrhosis can modulate bioecological system in the intestinal tract via prevention of the growth of pathogens, improvement to the mucosal layer, preservation of intestinal epithelia cells, and BT reduction. All of these mechanisms decrease portal hypertension due to inhibition of NO production followed by lowering plasma LPS (51). We performed further investigations to determine the effects of probiotics on cirrhosis than on other liver diseases. Data from several studies stated in Table 3 confirms the effects of probiotics.

**Table 3:** Several Animal and Clinical Trial Studies on Cirrhosis

	Participants/Duration	Treatment	Outcome	Reference
**Animal models**	Cirrhotic Rat induced by CCl4 / 10 d	*Lactobacillus johnsonii La1*	Intestinal enterobacteria and enterococci, bacterial translocation, MDA levels	(97)
**Animal models**	Cirrhotic Rat induced by CCl4 / 10 d	*Lactobacillus GG*	No effect on prevention of bacterial translocation	(98)
**Animal models**	Rat with acute liver injury / 8 d	*Bifidobacterium animalis NM2/ Lactobacillus acidophilus NMI/ Lactobacillus rhamnosus /Lactobacillus rhamnosus DSM 6594/ Lactobacillus plantarum DSM 9843*	Prevented alcohol-induced dysbiosis	(99)
**DB, PC**	36 patients / 6 m	*Lactobacillus.acidophilus, Lactobacillus bulgaricus, Bifidobacterium lactis, S. thermophiles*	The ammonia levels starting after 1 m/ no effect on liver enzyme	(93, 100)
**R, DB, PC**	65 patients / 6 m	*Lactobacilli*	Incidence of HE, hospital admission, plasma-ammonia level, serum bilirubin level	(101)
**R**	50 patients / 14 d	*Bifidobacterium, L. acidophilus *and* Enterococcus vs. Bacillus subtilis* and *Enterococcus faecium*	Bifidobacteriumcount, fecal pH, fecal and blood ammonia in both groups, endotoxin level only with B. subtilis and E. faecium	(69)
**PPT**	8 patients with HVPG ˃10 mmHg / 2 m	VSL # 3	In plasma endotoxin, serum TNF-α, plasma aldosterone	(102)
**RCT**	41 chronic liver disease / 14 d	*B. bifidus, L. acidophilus, L. bulgaricus*, and *S. thermophilus*	E. coli count, and intestinal flora imbalance, improvement in debilitation, food intake, abdominal distension, and ascitic fluid	(103)
**Pilot study**	39 patients / 48 d	*E. coli Nissle* (Mutaflor)	Improve intestinal colonization, endotoxin levels on day 42	(104)

Abbreviation: DB, double-blind; d, day; GGT; gamma glutamyl transferase; HE, hepatic encephalopathy; HVPG, hepatic venous pressure gradient; MDA, malondialdehyde; M, month; PC, placebo-controlled; PPT, perspective pilot study; RCT, randomized clinical trial; R, randomized; W, week.

#### 2.4.4. Primary Sclerosing Cholangitis

As far as we know, only two studies assessed the potential of the effects of probiotics on patients with PSC. In a pilot study designed by Vleggaar *et al.*, 14 patients with PSC with spontaneous inflammatory bowel disease were randomly administered Ecologic 641, the probiotic supplements with two *Bifidobacillus* and four *Lactobacillus* strains, or a placebo for three months. This treatment was performed as a double-blind manner. Results from this study demonstrated no improvement in pruritus, fatigue, and stool frequency. In addition, bilirubin, alkaline phosphatase, gamma glutamyl transpeptidase, aspartate aminotransferase, alanine aminotransferase, prothrombin, albumin, and bile salts levels were not significantly different between the probiotic and placebo groups (79).

In a case report, a thirteen year-old boy with PSC has been treated with standard therapy by prednisolone, salazosulfapyridine together with *Lactobacillus casei Shirota* as a probiotic. After two weeks, clinical symptoms, and results from laboratory tests had shown improvement (80). Overall, these reports could not confirm the beneficial effects of probiotics on the treatment or prevention of PSC disease, and further studies might be needed.

#### 2.4.5. Hepatic Encephalopathy

Evidence obtained from limited clinical trials performed on hepatic encephalopathy in human patients suggested that probiotic strains were able to decrease ammonia levels of serum, and improve neuropsychological symptoms (Table 4). It appears that a reduction of bacterial urease activity, alleviation of pH and ammonia absorption, and decreased intestinal permeability are the beneficial effects of probiotics for treating hepatic encephalopathy (58). It is unfortunate that there are no appropriate animal models to assess minimal hepatic encephalopathy (MHE). Also, in human trial studies, the use of high levels of viable strain is ridiculous; therefore, the number and quantity of doses increase. Another disadvantage to these studies is the inability to use urease producing bacteria because of their dangerous properties to humans. In addition, the sampling size of this trial is small, and approving probiotics as a useful treatment method for MHE is dubious.

**Table 4:** Multifarious Clinical Trials in Patients With Hepatic Encephalopathy

	Participants/Duration	Treatment	Outcome	Reference
**RCT**	40 patients/three 4 w with 2 weeks washout	*Enterococcus faecium S68 / lactolus*	Serum ammonia levels, improved various neurocognitive tests	(105)
**RCT**	50 patients / 30 d	Fermentable fiber or symbiotic 2000*vs*placebo	Fecal content of on-urease-producing *Lactobacillus species*, endotoxemia and blood ammonia levels, improvement in Child–Turcotte–Pugh class in 50% of patients	(106)
**RCT, PC, DB**	60 patients	*Bifidobacterium longum* with FOS*vs*placebo	Improving neuropsychological testing, serum ammonia levels	(107)
**RCT**	90 patients / 3 m	LOLA or lactolus or probiotic*vs*placebo	Improving blood ammonia levels	(108)
**RCT**	25 patients / 2 m	Yogurt	Significant rate of MHE reversal	(109)

Abbreviation: DB, double-blind; d, day; m, month; FOS, fructo-oligosaccharide; HE, hepatic encephalopathy; LOLA, L-ornithine L-aspartate; PC, placebo-controlled; RCT, randomized clinical trial; R, randomized; w, week.

#### 2.4.6. Viral Hepatitis

To investigate the responses to the probiotic supplements of patients with cirrhosis due to viral infections, Loguerico *et al*. treated 20 patients with HCV-related chronic hepatitis and 16 with HCV-related cirrhosis with VSL#3 (*Streptococcus thermophilus, Bifidobacterium berve, Bifidobacterium longum, Bifidobacterium infanti, Lactobacillus acidophilus, Lactobacillus plantarum, Lactobacillus casei*, and *Lactobacillus bulgaricus*) for four months. Aspartate aminotransferase, and alanin aminotransferase levels improved in two groups, and gamma glutamyl transferase improvement was observed only in HCV-related chronic hepatitis group (75). This area of liver damage requires more study to properly assess the benefits of probiotic therapy.

#### 2.4.7. Hepatocellular Carcinoma

Few studies were performed to assess probiotic effects on toxicity of aflatoxin in liver dysfunction and hepatocellular carcinoma. In a research performed by El-Nezami, diminution of aflatoxin concentration was observed in fecal samples after the administration of *Lactobacillus rhamnosus LC705* (81). In another five-week study, consumption of *Lactobacillus rhamnosus LC705* together with *Propionibacterium freudenreichii subsp. shermanii* led to lower AFB-N7 guanine in urine samples when compared to a placebo (63). In recent in-vivo, Kumar et al. studied gene expression changes induced by *Lactobacillus rhamnosus. GG* consumption in rats exposed to aflatoxin. Concomitant with lowering of the c-myc, bcl2, cyclin D1 and rasp21 expression in treated rats compared to control group, the frequency of tumors in liver was alleviated (64).

#### 2.4.8. Liver transplantation

Infection in a postoperative period is usually very prevalent in patients who have undergone liver transplantation. The most causative agents involved in these types of infections originate from the digestive tract (82). Generally, the results from four clinical trials performed on liver transplant patients described in Table 5, confirmed the inhibitory nature of probiotic consumption on postoperative infections.

**Table 5:** Clinical Trials Assessed Probiotics Roles on Postoperative Infections in Liver Transplantation

	Participants/Duration	Treatment	Outcome	Reference
**RCT, PC**	95 recipients	*Lactobacillus plantarum* and fermentable fiber, a heat-inactivated *Lactobacillus plantarum *and fiber, or selective intestinal decontamination	Duration of antibiotic therapy and hospital stay and intensive care unit stay, postoperative bacterial infections incidence	(82)
**R, DB, PC**	66 recipients / 15 d	*P. pentosaceus Leuconostoc mesenteroides, L. paracasei ssp. paracasei F19, L. plantarum 2362 Vs.* fibers	Postoperative bacterial infections incidence, ↓duration of antibiotic therapy	(110)
**DB, PC**	25 children recipients / 2 m	*L. casei strain DN-114001 vs.* glucose	β-glucuronidase,β-glucosidase, and urease, changes in the intestinal microbiota	(111)
**R, PC**	50 recipients / 16 d	*B. breve, L. casei*, and galactooligosaccharides	Postoperative bacterial infections incidence	(112)

Abbreviation: DB, double-blind; d, day; m, month; PC, placebo-controlled; RCT, randomized clinical trial; R, randomized; w, week.

## 3. Conclusions

A major instigate of liver disease is an anomaly in the gut flora. A balanced and healthy gut prevents a high percentage of harmful liver conditions. Several studies had been performed on various diseases, have confirmed the positive influence of probiotic strains on pathophysiological symptoms. Probiotic administration is safe, inexpensive and a noninvasive strategy as compared to antibiotic therapy and surgery. The expanding usage of antibiotics has resulted in the emergence of drug-resistant strains which pose a serious threat to humankind survival. Furthermore, the probiotic therapy shows no severe side effects unlike antibiotic therapy. Although results from clinical trials performed on common liver diseases showed the positive effects of probiotics, there are two problems that limit the usage of probiotics as a routine therapy. Since functional mechanisms of probiotic are specific to strain, recognize special strains with the highest prophylactic, and preventive properties on liver disease may be required. Also, engineering probiotics for specific, desirable properties might be useful. Lastly, to confirm the viability of bacteriotherapy, more clinical trials in various countries with disparate races, ethnicity, and lifestyles would be required.
